# Effectiveness of a Multimodal Digital Psychotherapy Platform for Adult Depression: A Naturalistic Feasibility Study

**DOI:** 10.2196/10948

**Published:** 2019-01-23

**Authors:** Enitan T Marcelle, Laura Nolting, Stephen P Hinshaw, Adrian Aguilera

**Affiliations:** 1 Department of Psychology University of California, Berkeley Berkeley, CA United States; 2 Leonard Davis School of Gerontology University of Southern California Los Angeles, CA United States; 3 Department of Psychiatry University of California, San Francisco San Francsco, CA United States; 4 School of Social Welfare University of California, Berkeley Berkeley, CA United States; 5 Department of Psychiatry San Francisco General Hospital University of California, San Francisco San Francisco, CA United States

**Keywords:** cognitive therapy, depression, digital health, live chat, mHealth, mental health, text messaging, video, mobile phone

## Abstract

**Background:**

Although psychotherapy is one of the most efficacious and effective treatments for depression, limited accessibility to trained providers markedly limits access to care. In an attempt to overcome this obstacle, several platforms seeking to provide these services using digital modalities (eg, video, text, and chat) have been developed. However, the use of these modalities individually poses barriers to intervention access and acceptability. Multimodal platforms, comprising those that allow users to select from a number of available modalities, may be able to provide a solution to these concerns.

**Objective:**

We aimed to investigate the preliminary effectiveness of providing psychotherapy through a multimodal digital psychotherapy platform. In addition, we aimed to examine differential responses to intervention by gender, self-reported physical health status, and self-reported financial status, as well as how prior exposure to traditional face-to-face psychotherapy affected the effectiveness of a multimodal digital psychotherapy intervention. Finally, we aimed to examine the dose-response effect.

**Methods:**

Data were collected from a total of 318 active users of BetterHelp, a multimodal digital psychotherapy platform. Data on physical health status, financial status, and prior exposure to psychotherapy were obtained using self-report measures. Effectiveness was determined by the extent of symptom severity change, which was measured using the Patient Health Questionnaire at Time 1 (time of enrollment) and Time 2 (3 months after enrollment). Intervention dosage was measured as the sum of individual therapist-user interactions across modalities.

**Results:**

Depression symptom severity was significantly reduced after the use of the multimodal digital psychotherapy intervention (*P*<.001). Individuals without prior traditional psychotherapy experience revealed increased improvement after intervention (*P*=.006). We found no significant dose-response effect of therapy, nor significant differences in outcomes across gender, self-reported financial status, and self-reported physical health status.

**Conclusions:**

Users of BetterHelp experienced significantly reduced depression symptom severity after engaging with the platform. Study findings suggest that this intervention is equally effective across gender, self-reported financial status, and self-reported physical health status and particularly effective for individuals without a history of psychotherapy. Overall, study results suggest that multimodal digital psychotherapy is a potentially effective treatment for adult depression; nevertheless, experimental trials are needed. We discuss directions for future research.

## Introduction

Major depressive disorder is a commonly occurring condition [1,2] associated with a multitude of adverse health outcomes [3,4] and is projected to be the second leading cause of disability worldwide by the year 2020 [5,6]. Development and dissemination of efficacious and accessible treatment for the disorder are an area of increasing importance. Psychotherapy has been shown to be one of the most efficacious psychosocial treatments for depression [7,8] and works by teaching patients cognitive strategies that enable them to target and manage undesirable thoughts, habits, and emotions underlying presenting depressive symptoms [9]. Although the literature has demonstrated the need for psychotherapy in the successful treatment of depression [10-13], limited geographical access to trained professionals remains one of the most significant barriers to traditional face-to-face care [14]. Telemental health, or the use of digital technology to provide long-distance clinical mental health care, is a field rapidly growing in response to issues of access [15-18].

Multimodal digital therapy platforms, that is, platforms that offer multiple modes of digital communication, hold promise in overcoming persisting barriers to intervention access and acceptability. In the United States, 1 in every 4 adults is in need of counseling, although only 13.4% of adults report receiving services [19]. This treatment gap is seen around the world [20] and is driven by a combination of stigma surrounding mental health support-seeking behavior [21] and limited geographical access to providers [14]. Digital psychotherapeutic interventions have been shown to increase intervention reach by providing efficacious alternatives to traditional therapy. These digital interventions increase both ease of access and anonymity of service seeking. To date, digital therapy has largely centered around the internet- (eg, videoconference or chat) and mobile (eg, short message service [SMS] text messaging or phone)-based modalities.

Extant literature demonstrates marked demographic differences in modality access and acceptance. Internet-based interventions have been shown to be efficacious [22], with a 2009 meta-analysis of 12 studies finding a mean effect size of *d=* 0.41 for internet-based psychological treatments for adult depression [23]. This effect size was increased (*d*=0.61) after excluding standalone interventions and considering only those that included support or guidance from a therapist. While evidence is mixed [24], research suggests that increased guidance improves the efficacy of internet-based psychotherapy [25]. Although such interventions have been found to be efficacious, barriers to accessible, reliable, and consistent internet connection keep these interventions beyond the reach of traditionally underserved populations [18]. In response to this persisting digital divide, researchers have investigated internet-free, mobile phone- and SMS text message-based therapies as a way of continuing to increase access to care [26]. Mobile phone access is considerably more ubiquitous than internet access [15,27], and the development of mobile platforms has enabled mental health professionals to provide care to an even greater percentage of individuals in need. In addition, mobile phone-based psychotherapy for adult depression has been demonstrated to be efficacious [28], yet this workaround does not come without its own set of limitations. Specifically, younger adults feel more comfortable communicating and building relationships remotely [29], although older adults prefer communication modalities that can mirror more traditional, in-person interactions, such as video calls. A multitude of studies have examined and demonstrated the efficacy and effectiveness of single- or even dual-modality digital psychotherapy platforms [22,30-34], but we know of no existing work investigating the effectiveness of a multimodal platform. We propose a multimodal psychotherapy platform that allows users to choose from internet-based video or live chat and internet-free, mobile SMS text message or phone therapy interchangeably as a potential solution to this demographic variability in intervention usability and effectiveness.

In addition to the modality, other important predictors of digital psychotherapy outcomes include demographic characteristics as well as level of engagement. The existing literature in face-to-face psychotherapy has focused on differences in outcomes between men and women, as well as on differences across ages and socioeconomic status. Despite growing product innovation, the characteristics and demographics of populations for whom digital psychotherapies do and do not work remains unknown. Although evidence of gender effects on psychotherapy outcomes is mixed, research suggests that women report an increased symptom reduction after traditional psychotherapy [35-39]. Yet research on Web-based psychotherapy suggests that men report an increased symptom reduction compared with women. Given higher rates of the stigma surrounding therapy-seeking behaviors in men [40], it has been suggested that the anonymity afforded by Web-based platforms may be an explanation for this latter finding. Research examining the effect of age on psychotherapy outcomes also remains largely inconclusive. Some evidence suggests that younger clients may respond more quickly and report greater posttreatment improvement in psychotherapy, although older adults are more likely to adhere to treatment [7,41,42]. The literature focusing on broader socioeconomic predictors of traditional psychotherapy acceptance and response has focused on economic status, physical health status, and experience with therapy as key predictive variables, finding positive correlations between outcomes and these factors. We speculate that prior counseling experience and higher financial or health status will predict improved digital psychotherapy outcomes as well. Although a number of researchers have examined the effect of engagement, or dose-response effect of psychotherapy, in traditional settings [43], considerably fewer studies have examined this relationship in the context of digital psychotherapy [44-46]. We additionally aimed to examine the dose-response effect of our digital psychotherapy platform.

The aim of this feasibility study was to investigate the initial effectiveness of delivering psychotherapy via BetterHelp, a multimodal internet- and mobile-based psychotherapy service provider. Bowen et al [47] defined a feasibility study as any study aiming to “determine whether an intervention is appropriate for further testing.” In line with this definition, this naturalistic and quasi-experimental investigation aimed to examine individuals’ responses to BetterHelp to generate useful data to guide and justify future randomized controlled trials. Effectiveness studies are defined as those that investigate the extent to which an intervention does more good than harm when administered in a “real-word” setting, as opposed to in ideal and highly controlled conditions [48,49]. In line with our aim of investigating the effectiveness of a multimodal method of delivering psychotherapy for adult patients with depression, this work defined the intervention effectiveness as a significant reduction in the depressive symptom severity among individuals using BetterHelp. We hypothesized that engagement with BetterHelp will significantly reduce depression symptom severity. In addition, in this exploratory study, we aimed to investigate the ways in which multimodal digital psychotherapy outcomes vary by subpopulation (gender, age, financial and physical status, and prior therapy experience) as well as by the level of engagement.

## Methods

### Participants

In total, 318 BetterHelp clients (of whom 254, 79.9%, were females), recruited from a larger pool of active BetterHelp users, participated in this study. BetterHelp users are individuals aged ≥18 years seeking to improve their quality of life. Users aged <18 years or under the care of a legal guardian were excluded from BetterHelp participation. Furthermore, individuals with thoughts of hurting themselves or others, those in urgent crisis or emergency situations, those diagnosed with a severe mental illness or advised to be in psychological supervision or psychiatric care, and those required to undergo therapy or counseling either by a court order or by any other authority were also excluded. BetterHelp users who met the eligibility criteria to participate in this study were invited to participate. Inclusion criteria for this study included a self-endorsed primary concern of feelings of overwhelming sadness, grief, or depression. Study participants were excluded from participation if preintervention levels of depression fell below mild clinical significance, that is, a score of <5 on the Patient Health Questionnaire (PHQ-9) [50], or if they had not engaged with BetterHelp for a minimum of 90 days. Users with preintervention depression in the mild to severe range, (ie, PHQ-9 scores ≥5 and ≤27) were included (Textbox 1). A total of 1148 BetterHelp users met all the eligibility criteria and were invited to participate. Our observed response rate of 27.70% (318/1148) is comparable to response rates seen in other Web-based survey studies [51]. The racial and ethnic makeup of this sample, as well as comorbid mental or physical health concerns, is unfortunately unknown as the BetterHelp platform does not currently collect this information. Ages of participants meeting the eligibility criteria ranged from 19 to 72 (mean 33.27 [SD 11.29]) years. At baseline, of 318, 119 (37.4%) participants met the criteria for mild depression, 91 (28.6%) for moderate depression, 75 (23.6%) for moderately severe depression, and 33 (10.4%) for severe depression [50,52,53] (Table 1).

**Inclusion and exclusion criteria for study participation.**

**Inclusion criteria**

- Age ≥18 years
- BetterHelp user for at least 90 days
- Baseline Patient Health Questionnaire score ≥5 and ≤27
- Self-endorsed primary concerns of feelings of overwhelming sadness, grief, or depression

**Exclusion criteria**

- Age <18 years
- Under the care of a legal guardian
- Thoughts of hurting self or others
- Severe mental illness
- Advised to be in psychological supervision or psychiatric care, or required to undergo therapy or counseling either by a court order or by any other authority
- BetterHelp user for <90 days
- Baseline Patient Health Questionnaire score <5

**Table 1:** Preintervention and postintervention Patient Health Questionnaire (PHQ-9) scores by the diagnostic category (N=318).

PHQ-9 diagnostic category	PHQ-9 score	Preintervention, n (%)	Postintervention, n (%)
Minimal depression	0-4	0 (0)	63 (19.8)
Mild depression	5-9	119 (37.4)	141 (41.2)
Moderate depression	10-14	91 (28.6)	66 (20.8)
Moderately severe depression	15-19	75 (23.6)	40 (12.6)
Severe depression	20-27	33 (10.4)	18 (5.7)

### Measures

#### Patient Health Questionnaire

The PHQ-9 [50,52,53] is a 10-item, self-report measure inquiring about the presence of depressive symptoms in the previous 2 weeks. It is used in clinical practice to monitor depression symptoms and severity [54]. The measure probes how often a respondent has been bothered by specific problems, takes only a few minutes to complete, and is scored on a 4-point Likert scale ranging from 0 (Not At All) to 3 (Nearly Every Day). The scale has demonstrated high internal consistency (Cronbach alpha=.86 to .89) as well as excellent test-retest reliability (*r*=0.84) [52]. Participants completed this measure at baseline and follow-up.

#### Working Alliance Inventory-Short Revised

The Working Alliance Inventory-Short Revised (WAI-SR) [55] is a 12-item measure assessing the quality of the therapeutic relationship. This measure of therapeutic alliance assesses the following domains: (1) agreement on the tasks of therapy; (2) agreement on the goals of therapy; and (3) development of an affective bond. The measure has consistently demonstrated good reliability (alpha>.80) as well as good convergent validity (*r*>0.64); it was administered at follow-up to assess user rapport with therapist.

#### Prior Exposure to Therapy

Before beginning therapy, a binary measure was administered to assess prior exposure to psychotherapy. BetterHelp users were asked the question “Have you ever been in counseling or therapy before?” and probed to reply with either “Yes” or “No.”

#### Self-Reported Physical Health Status and Financial Status

At baseline, BetterHelp users were asked to rate their current physical health and current financial status. Responses were scored on a 3-point Likert scale ranging from Good to Poor.

### Procedure

#### Intervention Description

The BetterHelp psychotherapy platform is currently the largest multimodal digital psychotherapy platform available worldwide [56]. BetterHelp utilizes a preference-based approach in which users can use any and all combinations of text, video, chat, or phone communication over the course of psychotherapy, as they choose. BetterHelp procedures are as follows: before beginning therapy, clients are asked to complete questionnaires probing symptom levels, personal history, and motivation for seeking therapy. Although BetterHelp counselors vary in approach (ie, cognitive behavioral therapy, acceptance and commitment therapy, etc), each BetterHelp counselor is a required to have attained a PhD, PsyD, Marriage Family Therapist, Licensed Clinical Social Worker, Licensed Professional Counselor, or Licensed Master Social Worker-level license to practice. BetterHelp’s algorithm then matches clients with an available BetterHelp counselor who best fits their objectives, counselor preferences, and needs. Preferred modality of communication is not taken into account in BetterHelp’s algorithm, as BetterHelp users can utilize any form of communication at any time, and all BetterHelp counselors are required to make themselves available to provide therapy through the client’s chosen modality. After the match is made, BetterHelp provides client and counselor with a dedicated “room” in which all communication takes place. Video, live chat, and phone sessions require advanced scheduling, while SMS text message exchanges do not. Multimedia Appendices 1-4 display BetterHelp platform as well as its video and live chat invitation and interface.

#### Data Collection

After engaging with the BetterHelp platform for 3 months, users received a notification inviting them to participate in an ongoing research study. This 3-month time window was selected to mirror the existing dose-response research in psychotherapy, suggesting that >50% of patients are able to respond after 12.7 sessions of weekly psychotherapy [57]. BetterHelp users were allowed 2 weeks to respond to prompts inviting them to participate in research. Users who did not respond within the allotted 2 weeks were excluded from study participation. Respondents were asked to repeat the PHQ-9 as well as complete the WAI-SR. All respondents to the research invitation completed all questionnaires. BetterHelp data analysts sent relevant data to authors in a deidentified data file.

#### Data Analysis

All analyses were conducted in SPSS Version 25 (IBM Corp Released 2017. IBM SPSS Statistics for Macintosh, Version 25.0) and R version 2.13.1 (R Development Core Team (2011). To examine the effectiveness of delivering psychotherapy through a multimodal digital platform, we examined the percentage of users exhibiting established markers of clinical improvement, as determined by change in PHQ-9 scores [53]. We first examined the percentage of users demonstrating clinically significant improvement. We additionally examined the percentage of users demonstrating a partial response to intervention and the percentage of users qualifying as being in remission after intervention use. We then examined changes in the symptom severity among BetterHelp users from pre- to posttreatment using paired samples *t* test. Because of our study design (ie, comparing mean PHQ-9 scores of BetterHelp users across 2 time-points), we determined paired samples *t* test to be the most appropriate method of analysis [58]. Our study design and the nature of our dependent and independent variables led us to determine a one-way analysis of covariance (ANCOVA), covarying baseline symptom severity, as the most appropriate means testing the differential effects of age and gender on therapy outcomes. To examine the effect of financial status, health status, and prior exposure to therapy on treatment outcomes, we conducted a second one-way ANCOVA, again adjusting for baseline symptom severity. The effect of dosage on treatment outcomes was examined using probit dose-response regression, a method used across the extant dose-response literature to predict the amount or dose of treatment needed to achieve a desired response or effect [43].

### Ethics Statement

This study was conducted in accordance with the Institutional Review Board and Office for the Protection of Human Subjects at the University of California, Berkeley. All subjects were informed of the risks and benefits of participating in the study and gave electronic informed consent to participate via the BetterHelp platform. Participants were provided with access to the BetterHelp platform and were not additionally compensated or incentivized to participate in any way. The research study was approved by the Institutional Review Board at the University of California, Berkeley.

## Results

### Descriptive Statistics

Table 2 describes the sociodemographic characteristics (age, gender, health or financial status, and prior exposure to therapy) of the study sample. Overall, of 318 participants, 72 (22.6%) rated their financial status as poor, 167 (52.5%) as fair, and 49 (15.4%) as good. In addition, 24 (7.5%) participants rated their health status as poor, 165 (51.9%) as fair, and 118 (37.1%) as good; furthermore, 91 (28.6%) participants did not have prior exposure to counseling or therapy. Table 3 displays the modality usage in the BetterHelp user sample.

**Table 2:** Sociodemographic characteristics of BetterHelp users.

Characteristic		BetterHelp users, n (%)	
**Gender**			
	Female		254 (79.9)
	Male		64 (20.1)
**Age (years)**			
	18-34		200 (62.6)
	35-49		89 (27.7)
	>50		29 (9.1)
**Physical health status**			
	Poor		24 (7.5)
	Fair		165 (51.9)
	Good		118 (37.1)
**Financial status**			
	Poor		72 (22.6)
	Fair		167 (52.5)
	Good		49 (15.4)
**Prior counseling experience**			
	No		91 (28.6)
	Yes		216 (67.9)

**Table 3:** Modality usage of BetterHelp users.

Modality usage	BetterHelp user, n (%)
Short message service text message user	318 (100)
Live chat user	53 (16.7)
Phone user	37 (11.6)
Video user	1 (0.3)
Single modality user	236 (74.2)
Dual modality user	74 (23.3)
Tri modality user	7 (2.2)
All modality user	1 (0.3)

### Overall Effectiveness of BetterHelp

**Figure 1:**
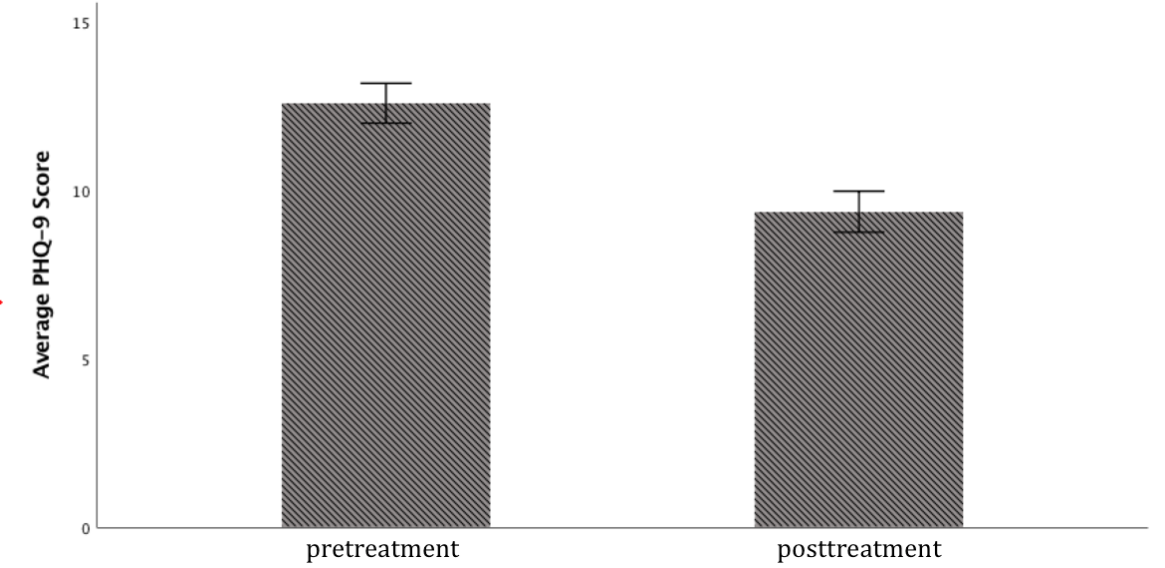
Overall Patient Health Questionnaire (PHQ-9) pre-post change. Error bars represent SEs.

In this study, of 318 participants, 120 (37.8%) demonstrated a clinically significant improvement and 194 (62%) demonstrated a partial response (as defined by at least a 5-point score reduction on the PHQ-9 and a postintervention score ≤9, respectively [53]) after engaging with BetterHelp for 3 months; in addition, 63 (19.8%) participants qualified as being in remission (as defined by a postintervention score <5) by Time 2. Paired samples *t* test results revealed a significant decrease in symptom severity posttreatment with effect size in the medium range (pretreatment: mean 12.57 [SD 5.35]; posttreatment: mean 9.36 [SD 5.51]; *t*_317_=10.80; *P*<.001, one-tailed; Cohen *d*=0.61; Figure 1). Mean PHQ-9 pretreatment scores reflected moderate levels of current depression, whereas mean posttreatment scores reflected mild levels of current depression, as determined by PHQ-9 clinical cutoffs [52]. As shown in Table 1, after using BetterHelp for 3 months, of 318 participants, 63 (19.8%) met the criteria for minimal depression, 141 (41.2%) for mild depression, 66 (20.8%) for moderate depression, 40 (12.6%) for moderately severe depression, and 18 (5.7%) for severe depression [52,53].

### Demographic Influence on Outcomes

A one-way ANCOVA examining the differential effects of age and gender on therapy outcomes, covarying baseline symptom severity, revealed no significant differences. Outcomes did not significantly differ across age (*F*_45,242_=0.98; *P*=.51) and gender (*F*_1,242_=.092; *P*=.76).

### Socioeconomic and Environmental Influence on Outcomes

**Figure 2:**
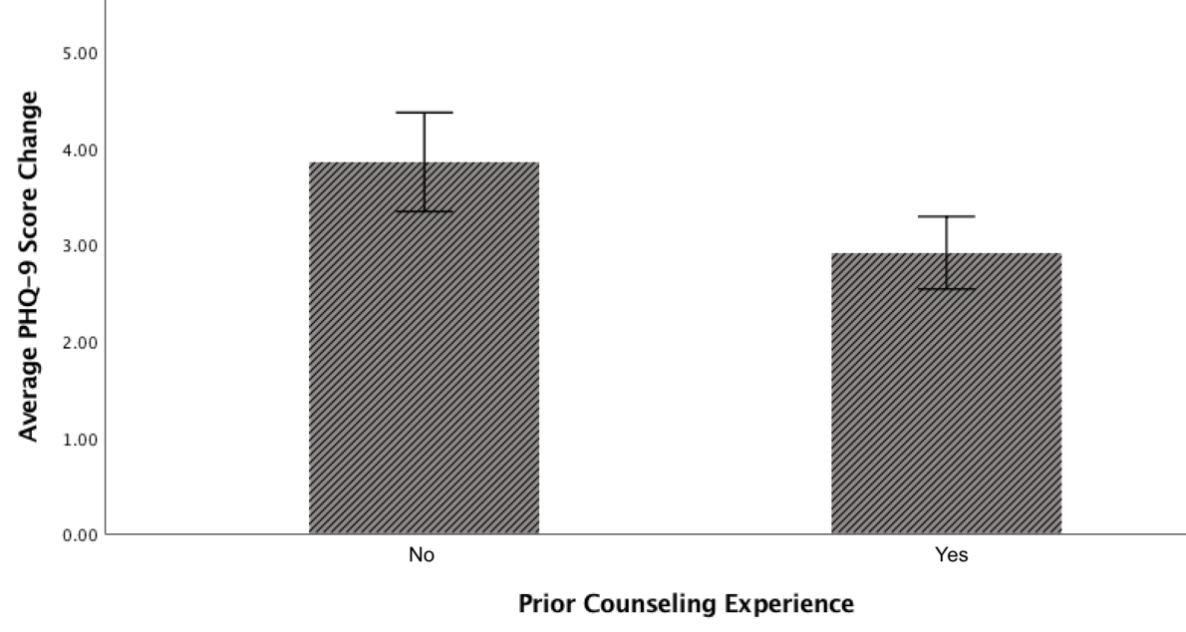
Difference in Patient Health Questionnaire (PHQ-9) score change by prior counseling experience status. Error bars represent SEs.

A one-way ANCOVA examining effects of financial status, health status, and prior exposure to therapy on treatment outcomes, covarying baseline symptom severity, revealed a significant effect of prior exposure to therapy on treatment outcome (*F*_1,260_=7.531; *P*=.006). Individuals with prior therapy exposure experienced significantly fewer gains after treatment compared with individuals without prior exposure (Figure 2). Treatment outcomes did not significantly differ across participant financial status (*F*_2,260_=1.563; *P*=.21) or health status (*F*_2,260_=1.575; *P*=.21).

### Dose-Response Effect

Treatment dosage was measured as the sum of individual therapist-user interactions across modalities (text message, phone, video, and live chat). Response was measured as a binary variable indicating the presence or absence of clinical improvement, as defined by a PHQ-9 score change of ≥5 points. Mean number of interactions in this study was 125.3 (SD 392.5). Following the methodology laid out by Howard et al in the probit model [59], the dose corresponded to the log of the number of total interactions, to reduce skew. Because participants who did not interact with BetterHelp were not included in the sample, taking the log of 0 was not a possibility. Results revealed no significant dose-response effect (*P*=.14), which was maintained after adding baseline severity into the model.

### Post-Hoc Analyses

To adjust PHQ-9 scores for regression to the mean, an ANCOVA in which the difference between baseline and posttreatment scores was regressed onto the mean-centered baseline score was used [58,60]. These additional analyses also revealed a significant mean difference (*P*<.001).

To assess the hypothesis that differences in treatment gains between individuals with and without prior therapy experience may be driven by differences in therapeutic alliance associated with prior therapy exposure, an independent samples *t* test was performed. Cronbach alpha for the 12 WAI-SR items was high (alpha=.946). The results revealed no significant effect of prior therapy experience on WAI-SR total scores (*P*=.55).

## Discussion

### Principal Findings

Our results indicate that multimodal digital psychotherapy may be an effective treatment for adult depression. Given our preliminary results demonstrating the effectiveness of this method of psychotherapy dissemination, we believe that multimodal digital psychotherapy may hold promise in overcoming some existing issues of psychotherapy access. Hence, continued research is needed. Users of a multimodal digital psychotherapy platform experienced significantly improved self-reported symptoms after engaging with BetterHelp, with 37.8% (120/318) users experiencing clinically significant improvement in depressive symptoms within 3 months. Moreover, no significant associations were found between changes in depressive symptoms and sociodemographic variables, including age, gender, and self-reported financial or physical health status. We suspect that increased accessibility and flexibility provided by a preference-based multimodal platform may be driving these latter findings [61,62]. Although this study cannot establish causality, our findings suggest that multimodal digital psychotherapy may be an effective solution to reduce existing barriers to accessible and preference-based digital psychotherapy.

Participants who had previously engaged in traditional face-to-face psychotherapy showed significantly less symptomatic improvement compared with those who had not. Among several potential explanations, we postulate that individuals with prior therapy experience may present with more chronic, complex, comorbid, or treatment-resistant forms of depression requiring a higher level of care [63-65]. Post-hoc analyses did not reveal any significant association between therapeutic relationship quality (as measured by the WAI) and prior therapy engagement, suggesting that this finding is not merely related to differences in therapist rapport and alliance linked to prior treatment exposure. Future research should seek to investigate differences in outcomes across diagnostic categories, taking into account commonly comorbid conditions known to influence response to treatment such as generalized anxiety disorder, attention deficit/hyperactivity disorder, and panic disorder. Baseline evaluation of the presence and severity of additional diagnoses may ensure that individuals with comorbid conditions are matched with an appropriately specialized therapist.

No general dose-response effect was detected in this study. Although this is not the first study to find nonsignificant dose-response effects of psychotherapy, we speculate that our existing measure of dosage may disguise existing dose-response effects, given that the quantity and significance of content shared in a single message may vary greatly by individual. Future research may seek to examine effects of word count as opposed to the message count, though due to user privacy concerns, researchers were not able to access these data at this time. Furthermore, the number of days spent interacting with the BetterHelp platform may prove to be a more valid measure of engagement than total interactions.

All study participants in this preference-based study utilized the SMS text message modality. A majority of participants utilized only one therapy modality over the course of the intervention, with only a single participant making use of all 4 modalities. We hypothesize that this pattern of use is driven by the increased convenience and flexibility provided by SMS text message-based therapy (ie, a previously scheduled appointment is not needed to utilize the SMS text message-based modality, whereas an appointment is needed to utilize live chat-, phone-, or video-based therapy). Future work will seek to test this hypothesis by obtaining qualitative data from participants regarding motivations behind modality choices, as well as to elucidate the added benefit of a multimodality psychotherapy platform (ie, using a randomized “text-only” vs “multimodal” design).

### Limitations

This exploratory investigation contains several limitations of note. First and foremost, we lacked a randomly assigned control group. Future investigations with appropriate controls, as well as a more comprehensive demographic and diagnostic screening, will enable a more valid approach. In addition, like many survey-based studies, the results of this work may be influenced by sample bias. It is possible that individuals who had notably positive or negative experiences with BetterHelp are those who chose to respond to our prompt to participate in research. We are further unable to investigate the effect of the type of psychotherapy provided (ie, cognitive behavioral therapy, acceptance and commitment therapy, etc) on outcomes. However, it is worth noting that the existing literature examining differential effects of different types of psychotherapies for depression does not suggest that psychotherapy type has a significant effect on outcomes in traditional psychotherapeutic settings [8]. In this study, financial and physical heath status were measured by self-report; such subjective indicators may not be fully valid. Future work may seek to utilize additional objective and sensitive measures of financial and physical health status to provide further insight. Finally, in this study, we lacked data on the potentially crucial moderator variables of race, ethnicity, and gender nonconformity.

### Conclusions

Major depressive disorder is a pervasive and debilitating condition from which many individuals are unable to recover due to lack of accessible and appropriate treatment. The existing literature has demonstrated the use of digital technology as a feasible solution to this growing and widespread dilemma. This study examined the effect of a multimodal digital psychotherapy platform for the treatment of depression in adults. We proposed that a multimodal platform through which users can dynamically select from multiple modes of digital communication throughout therapy may be an effective method of delivering psychotherapy to adults with depression. Our study results demonstrate the initial effectiveness of such a model, with users experiencing significant symptom reduction after the intervention. This feasibility study’s preliminary demonstration of such a platform’s effectiveness provides an important first step in understanding the potential benefits of such a model of mental health care delivery. Given these results, subsequent research will aim to investigate the unique benefits of a preference-based multimodal platform compared with in-person, single- or bimodal psychotherapy dissemination.

## Appendix

Multimedia Appendix 1 BetterHelp video session invitation.

Multimedia Appendix 2 BetterHelp live chat invitation.

Multimedia Appendix 3 BetterHelp live chat interface (user perspective).

Multimedia Appendix 4 BetterHelp messaging platform.

## Abbreviations

ANCOVA: analysis of covariance
PHQ-9: Patient Health Questionnaire
SMS: short message service
WAI-SR: Working Alliance Inventory-Short Revised
