# Supplementary figures and images for: Effectiveness of a Multimodal Digital Psychotherapy Platform for Adult Depression: A Naturalistic Feasibility Study
Source: JMIR Mhealth Uhealth. 2019 Jan 23;7(1):e10948. doi: 10.2196/10948 (PMC6364202; doi:10.2196/10948)

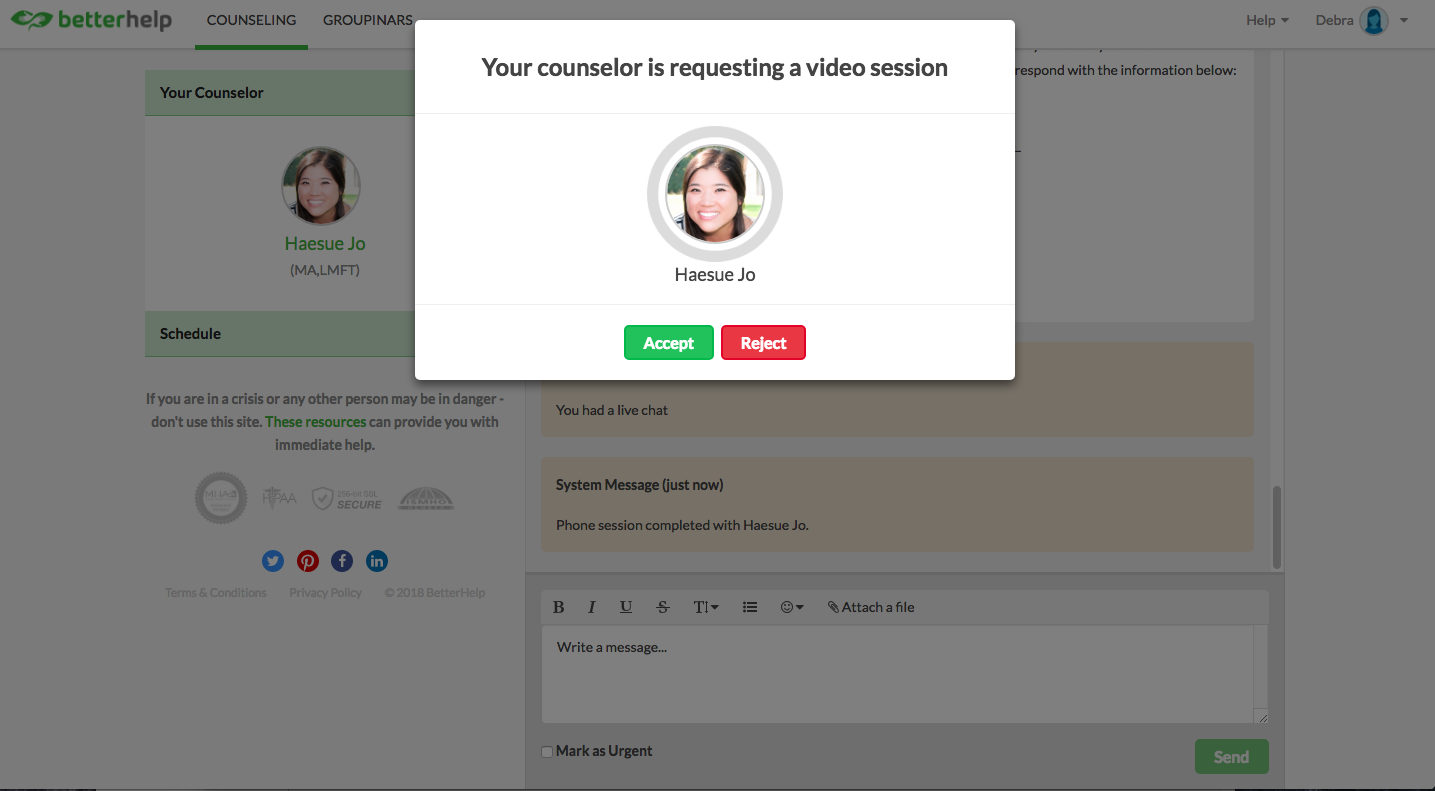

Supplement: Multimedia Appendix 1 [file mhealth_v7i1e10948_app1.png]

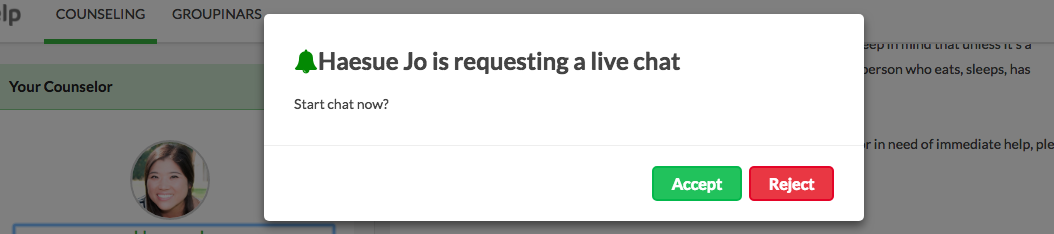

Supplement: Multimedia Appendix 2 [file mhealth_v7i1e10948_app2.png]

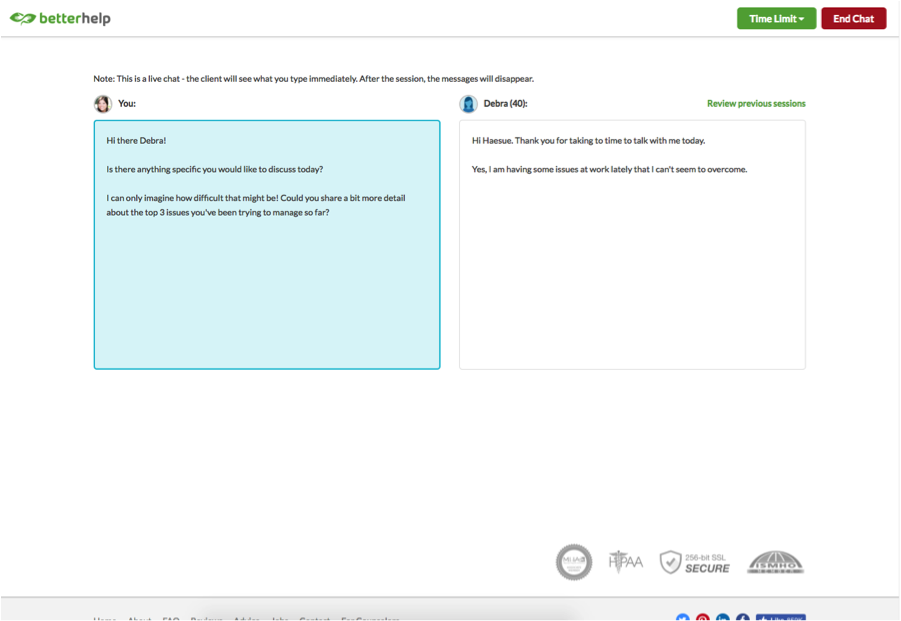

Supplement: Multimedia Appendix 3 [file mhealth_v7i1e10948_app3.png]

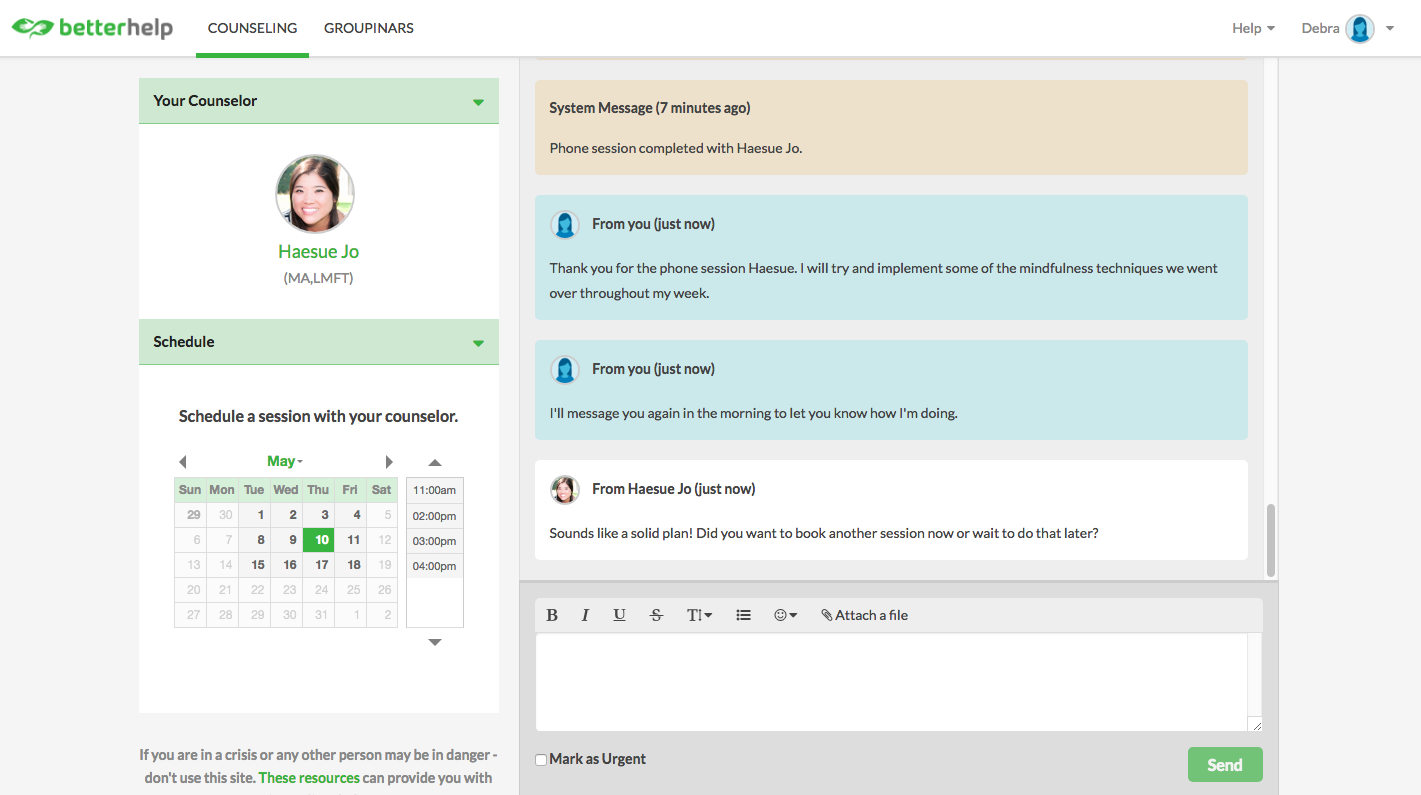

Supplement: Multimedia Appendix 4 [file mhealth_v7i1e10948_app4.png]
